# Constipation and risk of cognitive impairment and dementia in adults: a systematic review and meta-analysis

**DOI:** 10.3389/fneur.2025.1600952

**Published:** 2025-06-04

**Authors:** Qiyue Wang, Ting Yi, Xuan Jiang

**Affiliations:** Department of Gastroenterology, Beijing Tsinghua Changgung Hospital, School of Clinical Medicine, Tsinghua Medicine, Tsinghua University, Beijing, China

**Keywords:** constipation, colonic inertia, cognitive dysfunction, dementia, Alzheimer's disease, cognitive disability

## Abstract

**Introduction:**

It has been shown that constipation may have a close association with the occurrence of cognitive impairment (CI). This study was aimed at assessing the risk of CI in patients with constipation and exploring the interactions of constipation with other factors affecting CI.

**Methods:**

Embase, PubMed, Web of Science, and Cochrane were systematically searched to collect relevant literature for assessment of the association between constipation and CI. The included studies was subjected to quality assessment using the NIH quality assessment tool for observational cohort and cross-sectional studies. An odds ratio (OR) was calculated using a random effects model, and subgroup and sensitivity analyses were performed. Publication bias was assessed by the Egger's test and funnel plot, with a *P*-value of < 0.05 considered to indicate the presence of bias.

**Results:**

A total of 11 studies were included, including five retrospective studies and six cross-sectional studies, with 66,609 participants involved. The OR between constipation and cognitive impairment was 1.11 (95% CI: 1.03–1.20). Subgroup analysis showed that the OR between constipation and CI was 1.33 (95% CI: 1.12–1.58) in patients with Parkinson's disease and 1.05 (95% CI: 0.98–1.13) in patients without Parkinson's disease. The OR was 1.37 (95% CI: 1.07–1.74) in retrospective studies and 1.06 (95% CI: 0.98–1.15) in cross-sectional studies. The OR was 1.29 (95% CI: 0.92–1.80) in Europe and 1.12 (95% CI: 1.04–1.22) in Asia.

**Conclusion:**

The risk of CI was 1.11 times higher in constipated patients than in non-constipated patients. Constipation might significantly increase the risk of CI, especially in patients with Parkinson's disease.

**Systematic review registration:**

https://www.crd.york.ac.uk/PROSPERO/view/CRD42025630902, identifier: CRD42025630902.

## 1 Introduction

Constipation and cognitive impairment (CI) are common disorders in the geriatric population. As the world population ages, the incidence of both diseases continues to rise in older adults (1, 2). It has been shown that the incidence of constipation is high (about 20%) in older adults. Serious constipation is more common in older women, with an incidence two to three times higher than that in men of the same age (1). The effect of constipation is not limited to discomfort it causes, such as abdominal pain, abdominal distension, and difficult defecation, which can significantly reduce the ability of older adults to perform daily activities independently. In addition, CI (including mild CI and dementia) has a higher incidence in older adults aged 65 years and above as they age (3). Mainly manifesting as a decline in memory, attention and executive function, these problems seriously affect patients' daily life and increase the need for care. Studies have shown that CI patients usually have low quality of life, and the associated cost of care places a heavy financial burden on their families.

Although constipation and CI are seemingly separate health problems from each other, there is growing evidence that they may be closely related (4, 5) due to sharing the same risk factors such as age, gender, and economic status (3). Understanding the potential impact of constipation on CI, especially in the geriatric population, is of great importance for public health. Recently, it has been proposed that constipation may affect the degenerative process of cognitive function through an interaction with the brain-gut axis (6). As a bidirectional communication network between the brain and the gut, the brain-gut axis plays a key role in regulating cognitive function and gut health. It has been found that constipation may exacerbate cognitive decline by dysregulating gut microbiota, especially in the geriatric population (7). In addition, the association between constipation and CI may also play a role through inflammatory responses and neurotransmitter changes in the brain-gut axis. The two diseases interact to exacerbate the disease burden in older adults. Thus, the interaction between constipation and CI creates a greater health burden and caregiving stress for the geriatric population.

Although existing studies have indicated a certain association between constipation and cognitive impairment, such as the finding by Poplawska-Domaszewicz and scholars, who reported that early-onset Parkinson's disease (EOPD) patients carrying the GBA1 gene mutation may exhibit both constipation and cognitive decline. This suggests that constipation and cognitive impairment may be linked through shared genetic and pathological mechanisms (8). According to Nedelec et al. (9) and Leta et al. (10), constipation was considered to significantly increase the risk of developing CI. In contrast, Santos García et al. (11) showed that constipation did not significantly affect cognitive decline in the healthy population. These inconsistent conclusions may stem from differences in sample selection, study design, measurement methods, and assessment criteria across studies. To more fully understand the potential impact of constipation on CI, this study will also further explore other factors that may affect the association between constipation and CI, such as age, region of residence, and Parkinson's disease. These factors may affect the impact of constipation on CI to some extent, thus revealing the complexity and diversity of constipation as a potential risk factor.

## 2 Materials and methods

The aim of this meta-analysis (MA) was to assess the association of constipation with CI. This study was conducted in accordance with the PRISMA guidelines (12) and already registered on PROSPERO (CRD42025630902).

### 2.1 Data sources and search

A systematic search was conducted in PubMed, Cochrane, Web of Science, and EMBASE to identify relevant studies published until September 30, 2024. Search was not restricted by language, country or year of publication. The search strategy (Supplementary Table S1) was a combination of Medical Subject Headings (MeSH) and free-text terms, including “constipation,” “cognitive impairment,” and synonyms related to them. A comprehensive review of the included cross-sectional studies (CSSs) and cohort studies was conducted.

### 2.2 Eligibility criteria

Included studies were required to meet the following criteria: (1) cognitive impairment was clearly diagnosed using standardized tools such as the Mini-Mental State Examination (MMSE), Montreal Cognitive Assessment (MoCA), or Brief Interview for Mental Status (BIMS); (2) constipation was explicitly defined according to established criteria such as the Minimal Diagnostic Standards (MDS), Roman Criteria, or the Non-Motor Symptoms Scale (NMSS); (3) provision of odds ratios (ORs), relative risks (RRs), and hazard ratios (HRs) with their corresponding 95% confidence intervals, or provision of sufficient data to allow for the calculation of these statistics; (4) observational studies (including cross-sectional studies and cohort studies).

Exclusion criteria included (1) duplicate studies, i.e., exclusion of literature in which the same study was published multiple times or data were reused, so as to avoid data reuse; (2) language restriction, i.e., exclusion of literature in non-target languages (e.g., languages rather than English or Chinese); (3) type of literature, i.e., exclusion of non-primary studies such as reviews, commentaries, conference abstracts, case reports, and letters; (4) incomplete data, i.e., exclusion of literature that lacked key data or did not clearly reported findings; (5) inadequate quality of study design, i.e., exclusion of studies with obvious methodological flaws or quality scores below preset criteria; (6) non-extractable data, i.e., exclusion of studies that failed to provide key statistical indicators (e.g., OR, RR, HR, and their 95% confidence intervals) or studies with data formats incompatible for merge.

### 2.3 Data extraction

Two reviewers used a predesigned data extraction form (shown in Supplementary Table S1) for data extraction, and all disagreements were resolved by discussion. Information extracted included: the first author's name, year of publication, study design, study site, sample size, gender ratio, age, method for identifying constipation, adjusted risk estimates with corresponding 95% confidence intervals, and other relevant study characteristics. For studies that did not report effect sizes or confidence intervals, we estimated ORs by calculating the total number of participants in the constipated patients and non-constipated patients and the incidence of CI (13).

### 2.4 Quality assessment

In this MA, we used the quality assessment tool provided by the National Institutes of Health (NIH) to score five cohort studies (CSs) and six CSSs. The NIH quality assessment tool is a widely used standardized assessment tool designed to help researchers systematically assess the quality of studies, especially in terms of sample selection, data collection, and bias control. The tool provides appropriate assessment checklists based on different study designs (e.g., CSs and CSSs) to ensure scientific and consistent quality assessment.

### 2.5 Statistical analysis

Given the low incidence of cognitive impairment, the HR and RR were considered equivalent to the OR in statistical analysis for uniform interpretation and application. Heterogeneity was assessed by the statistic *I*^2^ (14). The *I*^2^ value was used to quantify heterogeneity among pooled estimates. A higher *I*^2^ value indicated higher heterogeneity. When the *I*^2^ value was more than 50% and the *P*-value was < 0.05, a random effects model would be used for analysis, otherwise, a fixed effects model would be used. Subgroup analysis would be conducted by study type, region, age, and presence of Parkinson's disease. In addition, sensitivity analysis would be conducted by removing studies one by one and recalculating the OR to assess the potential impact of individual studies on the overall results. In this study, a funnel plot was used to visually assess the symmetry of publication bias (PB), while an Egger's test was used to quantitatively analyze the relationship between effect sizes and standard errors, so as to comprehensively assess PB in the included studies. All results were considered statistically significant when *P* < 0.05. All analyses were performed using Stata 17.0.

## 3 Results

### 3.1 Search results

**Figure 1:**
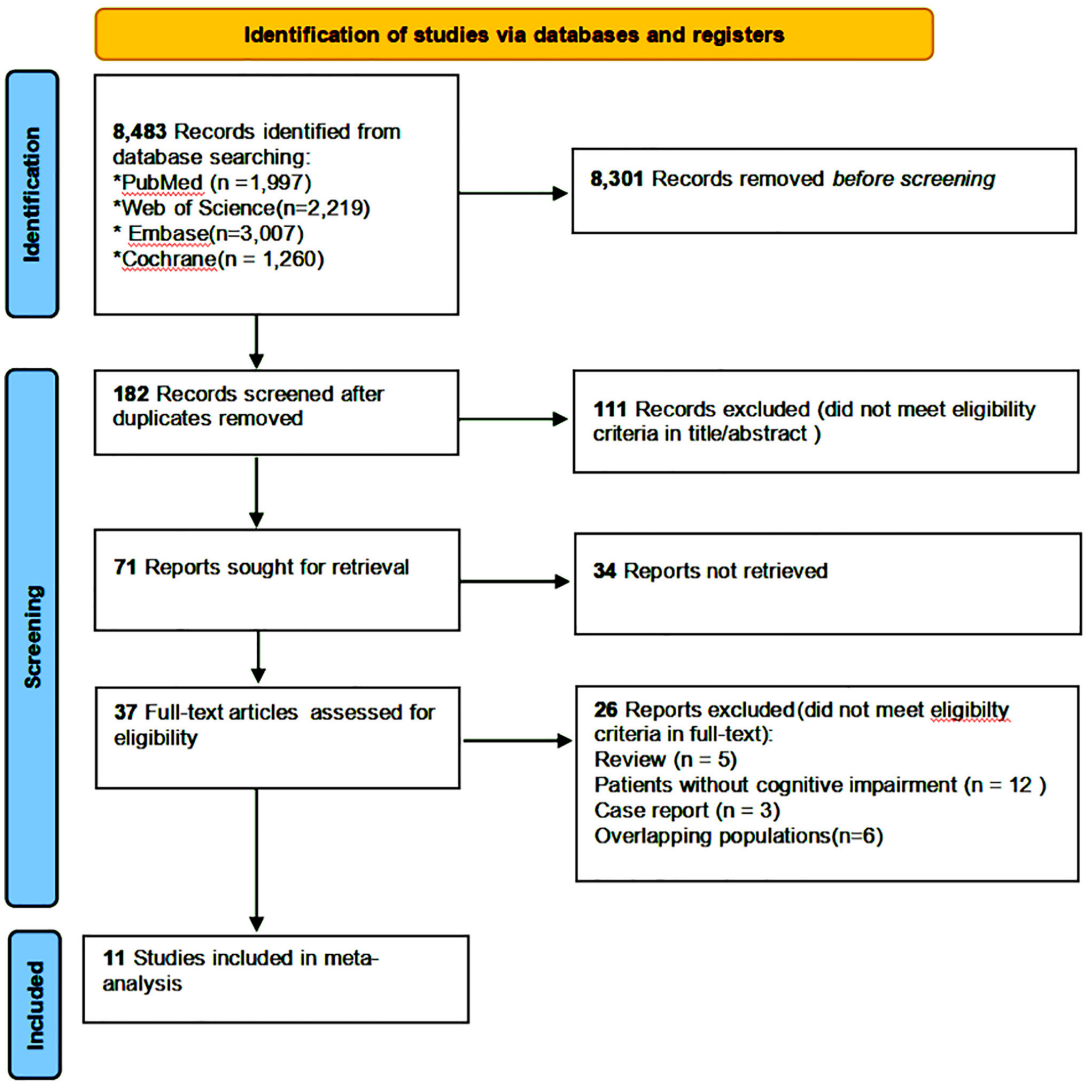
Flow diagram showing the identification and selection of studies on the association between constipation and CI.

A total of 8,483 studies were retrieved from the specified databases according to the planned search strategy. Of them, 463 that potentially met the inclusion criteria were left after removal of duplicates and screening by title and abstract. After a full-text review, 11 studies (six CSSs and five CSs) were finally included for MA (shown in Figure 1) (4, 11, 15–23).

### 3.2 Study characteristics

The baseline characteristics of the included studies were summarized in Table 1. Six CSSs and five CSs were included in this study, with a total of 66,609 participants (including 22,645 ones in the CSSs and 43,964 ones in the CSs). The studies were published in Europe (five studies), the United States (one study), China (four studies), and Japan (one study), and between 2016 and 2024. The methods used to identify constipation varied across the studies. They were usually the Rome IV criteria, Movement Disorder Society-sponsored revision of the Unified Parkinson's Disease Rating Scale (MDS-UPDRS) or ICD-10 code, as well as self-report questionnaire (e.g., frequency of bowel movements). The results of quality assessment showed that all the five CSs were scored 11 and all the six CSSs were scored 9, indicating that the studies were of high quality and highly reliable.

**Table 1:** Characteristics of the study population.

**Author**	**Year**	**Country**	**Design**	**Constipation group members**	**Non-constipated group members**	**Age**	**Gender (male/female)**
Lamas	2016	Sweden	Cross-sectional	1,980	959	76.3–91.9	941/1,998
Allen	2017	America	Cross-sectional	3,278	3,278	>18	1/1
Picillo	2020	Italy	Retrospective cohort study	322	0	67–68	209/113
Camacho	2021	England	Retrospective cohort study	152	313	57.5–78.9	283/182
Leta	2021	33 centers including the United States, Europe, Israel, and Australia	Retrospective cohort study	137	286	54.01–71.09	277/146
Sun	2021	China	Cross-sectional	87	79	54.09–74.26	90/76
Garcia	2022	Spain	Retrospective cohort study	198	301	53.73–71.05	296/203
Wang	2022	China	Cross-sectional	1,785	9,958	68–74	5,167/6,576
Shimizu	2023	Japan	Retrospective cohort study	10,032	32,223	60.2–62.0	19,396/22,859
Huang	2024	China	Cross-sectional	131	658	62.2–77.0	328/461
Su	2024	China	Cross-sectional	234	218	51.6–68.6	201/251

### 3.3 Constipation and risk of CI

**Figure 2:**
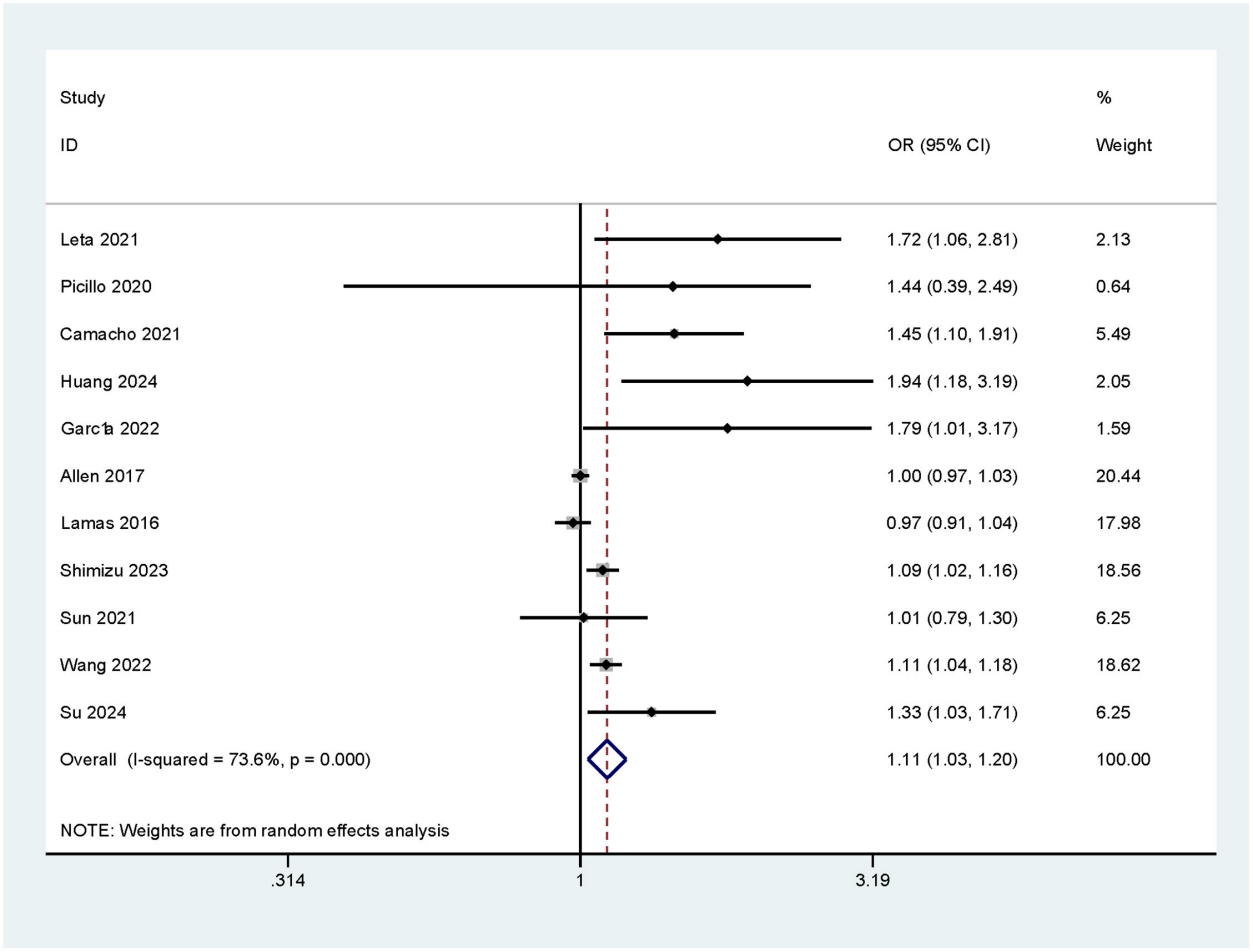
The risk of CI in patients with constipation in different studies.

There was a significant positive association of constipation with CI. A total of 11 relevant studies were included, with an *I*^2^ value of 73.6%, which suggested high heterogeneity. Further analysis by a random effects model showed that constipation significantly increased the risk of CI (OR = 1.11, 95% CI: 1.03–1.20). This result suggests that constipation may be a potential risk factor for the occurrence of CI (as shown in Figure 2).

#### 3.3.1 Impact of Parkinson's disease (PD) on the risk of CI caused by constipation

**Figure 3:**
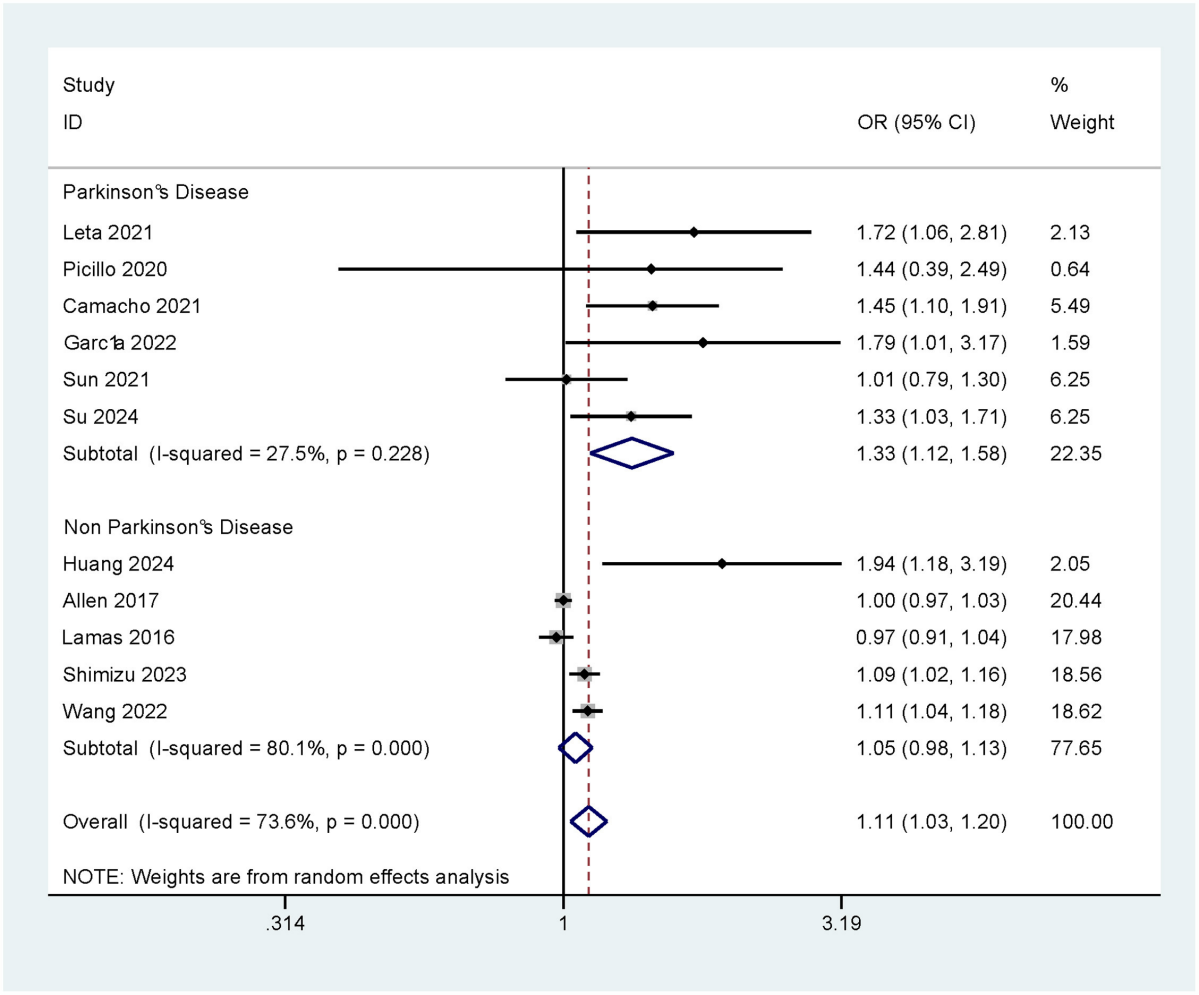
OR and 95% CI for CI caused by constipation in PD.

Subgroup analysis of the study population was conducted according to the presence/absence of PD, so as to further explore the association between PD and the risk of CI due to constipation. The results showed that constipation significantly increased the risk of developing CI in patients with PD (OR = 1.33, 95% Cl: 1.12–1.58). In contrast, in patients without PD, the association between constipation and the development of CI was not significant (OR = 1.05, 95% CI: 0.98–1.13; as shown in Figure 3).

#### 3.3.2 Constipation and risk of CI in different study types

Two study designs were included in this MA: CSSs and CSs. All of them assessed the association between constipation and the risk of developing CI. The results of the CSSs showed (4, 18–22) no significant association between constipation and CI (OR = 1.06, 95% CI: 0.98–1.15; Supplementary Figure S1).

In contrast, the CSs showed that constipation significantly increased the risk of developing CI (OR = 1.37, 95% CI: 1.07–1.74; Supplementary Figure S1).

#### 3.3.3 Risk of cognitive impairment associated with different constipation assessment criteria

**Figure 4:**
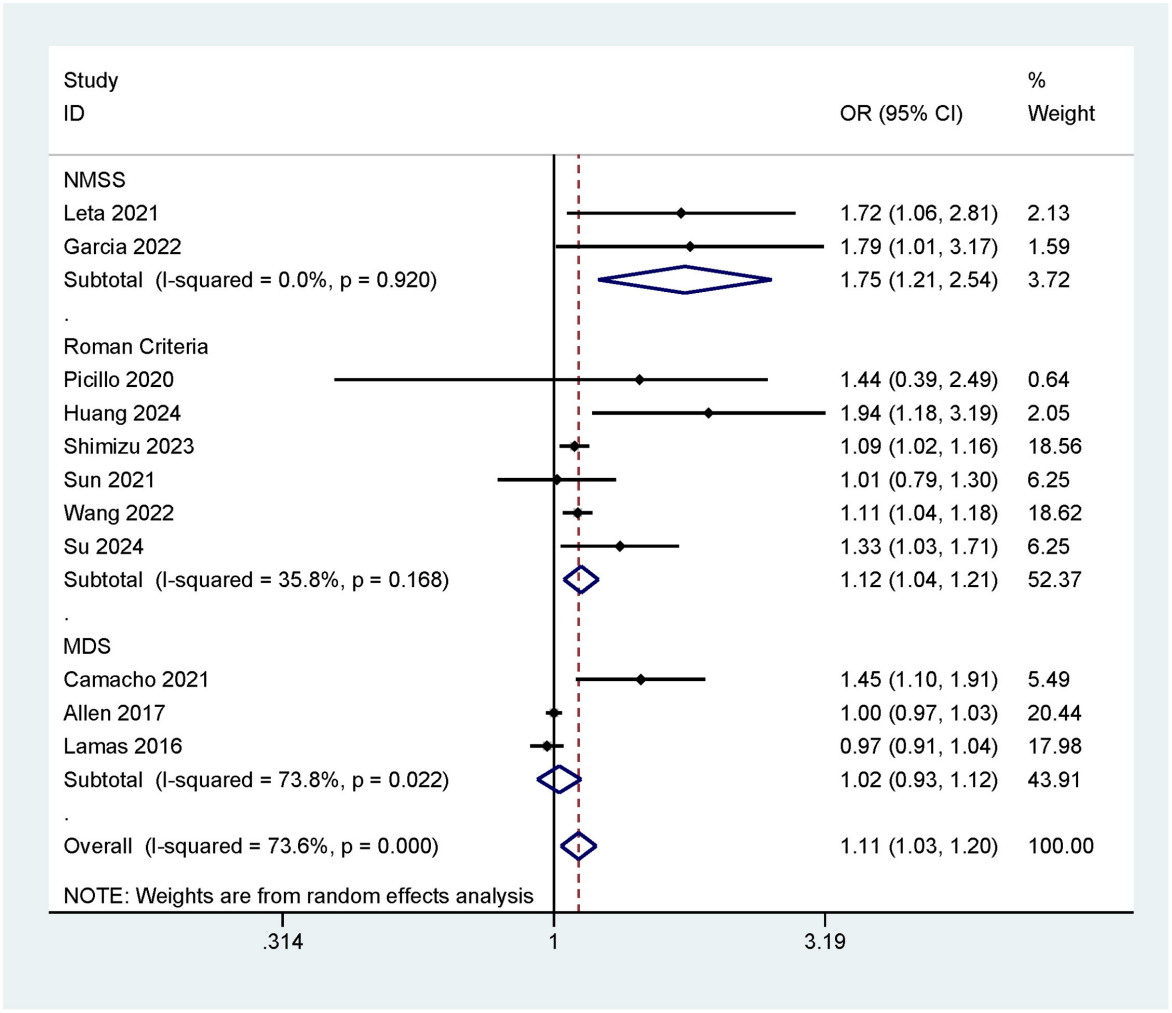
OR and 95% CI for CI in patients with different constipation assessment criteria.

This meta-analysis employed three constipation assessment criteria (MDS/Roman Criteria/NMSS) to evaluate patients' defecation status. To compare the association between constipation and the risk of cognitive impairment under different constipation criteria, subgroup analyses were conducted based on these different criteria. The results indicated that when assessed using the NMSS criteria, constipation was significantly associated with an increased risk of cognitive impairment (OR = 1.75, 95% CI: 1.21–2.54; Figure 4). When evaluated using the Roman Criteria, constipation also showed a positive correlation with cognitive impairment (OR = 1.12, 95% CI: 1.04–1.21; Figure 4). However, when assessed using the MDS criteria, there was no significant association found between constipation and cognitive impairment (OR = 1.02, 95% CI: 0.93–1.12; Figure 4).

#### 3.3.4 Risk of cognitive impairment associated with different cognitive assessment scales

**Figure 5:**
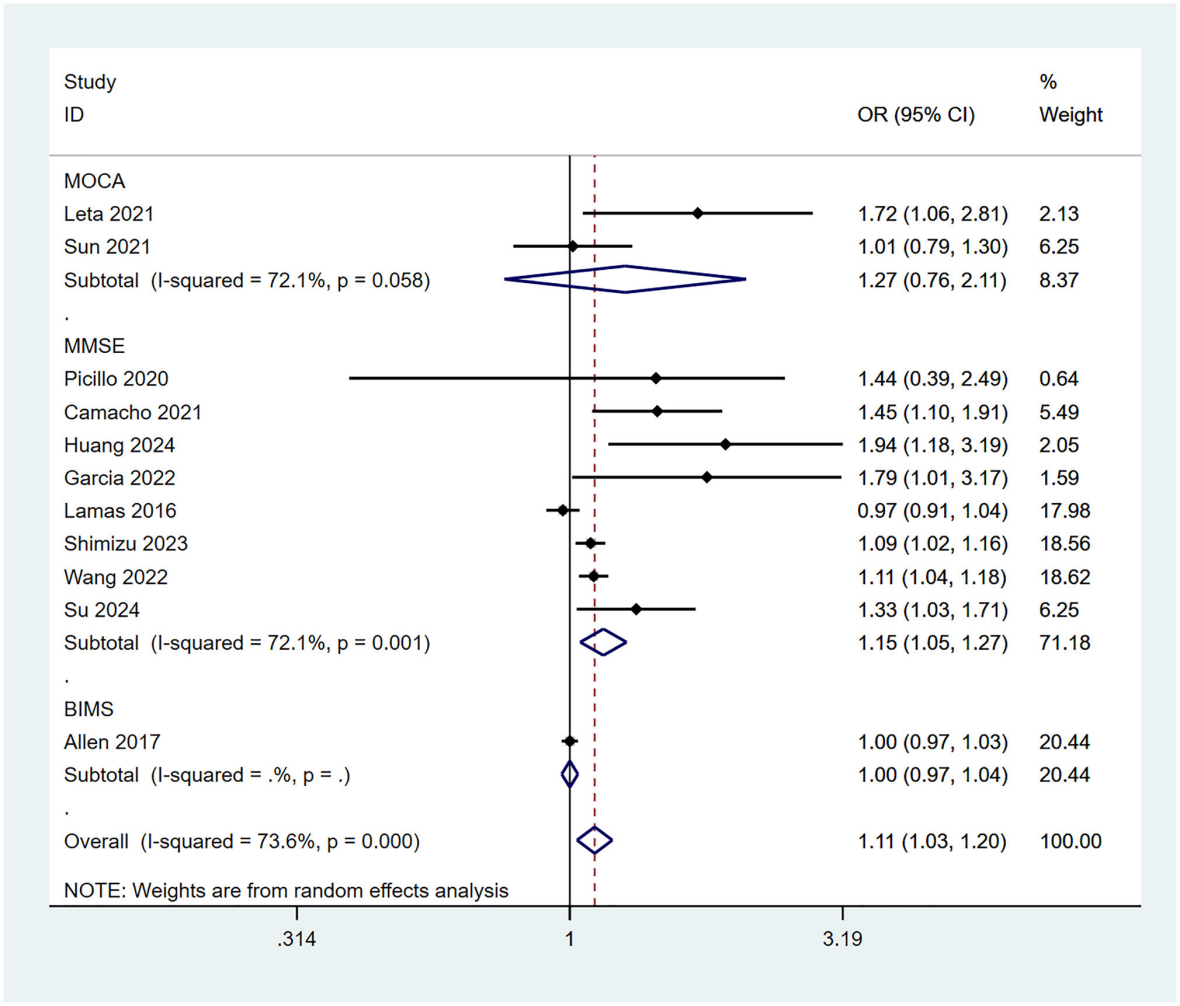
OR and 95% CI for CI in patients with different cognitive assessment scales.

This meta-analysis employed three cognitive assessment scales (MMSE/MoCA/BIMS) to evaluate patients' cognitive impairment. To compare the association between constipation and the risk of cognitive impairment using different assessment tools, subgroup analyses were conducted based on the type of scale. The results indicated that when assessed using the MMSE scale, constipation was significantly associated with an increased risk of cognitive impairment (OR = 1.15, 95% CI: 1.05–1.27; Figure 5). However, when evaluated using the MoCA (OR = 1.27, 95% CI: 0.76–2.11; Figure 5) or BIMS (OR = 1.00, 95% CI: 0.97–1.03; Figure 5), no significant association was found between constipation and cognitive impairment.

#### 3.3.5 Impact of sex on the risk of cognitive impairment caused by constipation

To further investigate the relationship between sex and the risk of cognitive impairment caused by constipation, we conducted a subgroup analysis based on the sex composition of the study populations. The results showed that in both male-predominant and female-predominant populations, constipation was positively associated with the occurrence of cognitive impairment (OR = 1.20, 95% CI: 1.02–1.41; OR = 1.16, 95% CI: 1.00–1.34, respectively). Notably, the positive association was more pronounced in male-predominant populations (Supplementary Figure S2).

#### 3.3.6 Impact of age on the risk of cognitive impairment caused by constipation

To further investigate the relationship between age and the risk of cognitive impairment caused by constipation, we stratified the study populations into different subgroups according to age: 50–60, 60–70, 70–80, and 80–90 years. The analysis results indicated that in the 50–60 and 60–70 years age groups, constipation was significantly associated with an increased risk of cognitive impairment (OR = 1.33, 95% CI: 1.03–1.71; OR = 1.32, 95% CI: 1.09–1.60, respectively; Supplementary Figure S3). However, no significant association was observed in the 70–80 and 80–90 years age groups.

#### 3.3.7 Risk of CI with constipation in different regions

To further explore the effect of regions on study results, subgroup analysis by region was conducted. The results showed that constipation increased the risk of CI in four studies from Europe (11, 16, 17, 20) (OR = 1.29, 95% CI: 0.92–1.80; Supplementary Figure S4).

In addition, analysis of five studies from Asia (4, 18, 21–23) showed that constipation increased the risk of CI (OR = 1.12, 95% CI: 1.04–1.22; Supplementary Figure S4).

In contrast, only one study from North America (19) was included, and the results showed no significant association between constipation and the development of CI (OR = 1.00, 95% CI: 0.97–1.04; Supplementary Figure S4).

### 3.4 Sensitivity analysis

Sensitivity analysis was conducted to further verify the robustness of our conclusion. The results showed that the overall OR value remained stable after the removal of any individual study, with no significant fluctuation in the 95% CI. This indicated that removal of individual studies did not significantly affect the overall effect estimate, thus verifying the robustness and reliability of our findings (Supplementary Figure S5).

### 3.5 Test for PB

The funnel plot shows that all included studies fell within the 95% CI, and the funnel plot look perfectly symmetric, indicating that no significant PB was observed (as shown in Supplementary Figure S6). Further analysis using an Egger's test also found the absence of significant PB, with *P* = 0.244.

## 4 Discussion

This study systematically assessed the association of constipation with CI through an MA of 11 studies including 66,609 patients. The results showed that constipation increased the risk of developing CI (OR = 1.11, 95% CI: 1.03–1.20). Subgroup analysis further found that constipation more significantly increased the risk of developing CI in patients with PD (OR = 1.33, 95% CI: 1.12–1.58).

Constipation is significantly associated with an increased risk of cognitive impairment, which is consistent with the findings of Poplawska-Domaszewicz et al. They discovered that GBA1 gene mutations accelerate the pathological spread of α-synuclein through the gut-brain axis, leading to the co-occurrence of autonomic dysfunction (such as constipation) and cognitive decline in PD patients. Additionally, Leta et al. also found a significant association between constipation and the risk of cognitive impairment. This association may be closely linked to interactions within the gut-brain axis, changes in gut microbiota, and vagus nerve signaling (24, 25).

In addition, the association between Parkinson's disease and the risk of cognitive impairment due to constipation was more significant. Cognitive impairment associated with PD can be categorized into three main clinical phenotypes: cholinergic type (characterized by basal forebrain degeneration, hyposmia, and rapid cognitive decline), noradrenergic type (associated with locus coeruleus damage, presenting as Rapid Eye Movement Sleep Behavior Disorder (RBD), anxiety, and attention deficits), and dopaminergic type (primarily involving executive dysfunction) (26). In patients with Parkinson's disease, there was a significant positive association between constipation and the development of cognitive impairment (OR = 1.33, 95% CI: 1.12–1.58). In contrast, in patients without Parkinson's disease, the association between constipation and the development of cognitive impairment was not significant (OR = 1.05, 95% CI: 0.98–1.13). The underlying mechanism is closely related to the cholinergic phenotype: the deposition of α-synuclein in the enteric nervous system and the dorsal nucleus of vagus nerve triggers microbial dysbiosis (such as reduced short-chain fatty acids) and neuroinflammation through the gut-brain axis. This, in turn, accelerates the degeneration of cholinergic neurons, leading to stepwise cognitive decline. Meanwhile, the noradrenergic phenotype may play a synergistic role. In patients with constipation combined with RBD or anxiety, lesions in the locus coeruleus can further promote the trans-synaptic spread of α-synuclein, resulting in the formation of Lewy bodies in the limbic system and cortex. Lewy bodies are a typical pathological hallmark of Parkinson's disease and other α-synucleinopathies (27). They consist of aggregated α-synuclein. Their accumulation in neurons may induce inflammation and accelerate the development of neurodegenerative lesions. This process reveals why constipation in PD patients further exacerbates the risk of cognitive impairment (28). Additionally, PD patients with comorbid constipation often have other non-motor symptoms such as depression, anxiety, and sleep disorders. These symptoms may interact through the cholinergic-noradrenergic pathways, potentially exacerbating cognitive impairment, emotional disturbances, and sleep quality issues. Collectively, they may contribute to a greater decline in brain health and cognitive function (29).

The differences in the ability of various cognitive assessment scales to detect the association between constipation and cognitive impairment may be related to variations in their sensitivity across cognitive domains, as well as to insufficient sample sizes in individual studies. In this meta-analysis, eight studies that used the MMSE to assess cognitive impairment showed that constipation may be a potential risk factor for cognitive decline (OR = 1.15, 95% CI: 1.05–1.27). Patients with constipation often exhibit abnormalities in the gut-brain axis and dysbiosis of the gut microbiota, which may exacerbate hippocampal damage and memory decline through inflammatory factors such as IL-6 and Tumor Necrosis Factor-alpha (TNF-α) (30). The MMSE evaluates cognitive function through questions regarding orientation in time and place, word repetition, and other tasks, making it particularly sensitive to declines in orientation and memory (features commonly associated with neurodegenerative diseases) and thus more likely to reveal the potential association between constipation and cognitive impairment. In contrast, the MoCA and BIMS are more sensitive to executive function and abstract thinking but lack sufficient assessment of memory and orientation. The results of two studies using the MoCA scale (OR = 1.27, 95% CI: 0.76–2.11) and one study using the BIMS scale (OR = 1.00, 95% CI: 0.97–1.03) did not show a statistically significant association between constipation and cognitive impairment. Furthermore, limited sample sizes may amplify differences between the scales. Future studies with larger sample sizes should employ multiple cognitive assessment tools concurrently to compare the risk of cognitive impairment associated with constipation across different scales.

Among the three constipation assessment criteria included in this study, both the Roman Criteria (OR = 1.12, 95% CI: 1.04–1.21) and the NMSS (OR = 1.75, 95% CI: 1.21–2.54) showed a positive correlation between constipation and the risk of cognitive impairment. This suggests that constipation may contribute to an increased risk of neurodegeneration and further cognitive decline through pathways such as the gut-brain axis, changes in gut microbiota, and abnormal activation of the vagus nerve pathway (31). Notably, the NMSS demonstrated a higher effect size compared to the Roman Criteria, which may be attributed to its primary use in assessing constipation in patients with neurodegenerative diseases. These patients typically have higher levels of α-synuclein deposition in the brain, which can retrogradely propagate via the vagus nerve, enhancing gut-brain axis interactions and directly exacerbating cognitive decline. On the other hand, the MDS criteria mainly capture short-term constipation symptoms, which might explain why it did not show a significant association between constipation and cognitive impairment.

Subgroup analysis results in populations with different sex ratios showed that both in male-predominant and female-predominant groups, constipation was positively associated with the occurrence of cognitive impairment (OR = 1.20, 95% CI: 1.02–1.41; OR = 1.16, 95% CI: 1.00–1.34, respectively). Notably, the positive association was more pronounced in male-predominant populations. This may be related to the neuroprotective effects of estrogen, which activate the PI3K/Akt pathway, enhancing the contraction of colonic smooth muscle cells and inhibiting the nuclear translocation of the inflammation gene transcription factor NF-kappaB, thereby reducing the production of inflammatory factors (32). In contrast, androgens can increase intestinal epithelial permeability, leading to increased leakage of brain-derived neurotrophic factor (BDNF) (33), resulting in impaired synaptic plasticity and reduced neuronal density, thus further exacerbating the detrimental impact of constipation on cognitive function. These findings suggest that sex should be considered as part of the cognitive assessment for patients with constipation. For male patients with constipation, it may be advisable to initiate cognitive screening at an earlier stage.

Interestingly, subgroup analysis did not confirm that advancing age intensifies the association between constipation and cognitive impairment. Instead, it revealed a positive correlation in the 50–70 age group (OR = 1.32–1.33), while this association was not significant in individuals over 70 years old. This finding challenges the traditional notion that “the older the age, the higher the vulnerability of the gut-brain axis” (34), suggesting that the interaction patterns between the gut and brain during aging may follow a “dynamic equilibrium” rather than a “linear deterioration.” In very older individuals (>70 years), constipation is more likely to be secondary to organic diseases (such as Parkinson's disease, diabetic gastroenteropathy), and standardized treatments for these conditions (e.g., dopaminergic medications, blood glucose control) may offset the negative impact of gut disorders on cognition. Conversely, constipation in relatively younger elderly individuals is often due to functional bowel motility insufficiency, more directly reflecting an imbalance in the gut-brain axis. On the other hand, even though cumulative gut-derived inflammatory factors (such as IL-6, TNF-α) can breach the blood-brain barrier and cause reversible neural damage, the human brain can adapt to structural degeneration through neural compensation (35). Neurodegeneration itself does not necessarily lead to continuous cognitive decline; chronic low-grade inflammation in older individuals may have reached a steady-state threshold where additional stimuli are unlikely to further exacerbate cognitive decline.

Geographical differences are also one of important findings from this study. There were differences in the definition and impact of constipation in different regions. We found that the risk of CI was higher in patients with constipation in Europe than in Asia, which may be related to local medication use (36), dietary habits (high-fat/low-fiber foods) (37), and psychological factors such as high-functioning depression and loneliness (38, 39). In Europe, animal husbandry is well-developed, and the traditional dietary culture is based on meat. In addition, due to geographical and climatic conditions in Europe, agricultural crops such as potatoes, carrots, and cabbages are mainly grown, with a relatively low fiber content. A high-fat/low-fiber foods may induce the growth of Gram-negative bacteria to increase the production of lipopolysaccharide (LPS), which is able to activate the immune system via Toll-like receptor 4 (TLR4), triggering an inflammatory reaction, a chronic low-grade inflammation that can damage the nervous system and induce cognitive impairment (40). Also in the European population, there was a higher prevalence of loneliness and depression. These psychological factors may influence the occurrence of constipation and be significantly associated with the risk of CI (41). Secondly, we selected only commonly used biomedical and scientific electronic databases for literature search. Although we conducted a systematic literature search and screening, we cannot guarantee inclusion of all relevant studies. In addition, different definitions of constipation in different countries and regions may have influenced our findings.

This study provided insight into the impact of constipation on the risk of cognitive impairment through subgroup analysis based on different study designs (e.g., retrospective studies and cross-sectional studies), patient populations (e.g., patients with Parkinson's disease and patients without Parkinson's disease), as well as differences in sex, region, and assessment scales. Such analysis revealed heterogeneity of the association between constipation and cognitive impairment across different populations, thus providing more refined guidance for clinical decision-making. However, there are still some limitations in this study. First, there was high heterogeneity among the included studies. The results of the retrospective studies and cross-sectional studies may have been affected by methodological differences, especially the failure of the cross-sectional studies to reveal a causal association. Second, the relatively small sample sizes in some subgroups (such as studies from different regions and those using different cognitive assessment scales) may have led to a lack of stability in the results of analysis. In addition, despite the in-depth exploration of different population by subgroup analysis in this study, the impact of potential confounding factors on the results still cannot be ruled out. For instance, we did not systematically assess the potential moderating effects of medication use (such as anticholinergic drugs, and opioid formulationsc) and dietary patterns (such as dietary fiber intake, and probiotic supplementation) on the risk of constipation-related cognitive impairment. These factors might alter the strength of the association between constipation and cognitive dysfunction by influencing gut microbiota composition, gut-brain axis signaling, or neuroinflammatory processes. Future studies should incorporate variables such as medication history and dietary assessments to more comprehensively elucidate the mechanisms by which constipation, as a modifiable factor, affects cognitive function. Therefore, more longitudinal studies and multicenter studies with larger sample sizes are still needed in the future to further verify the association between constipation and cognitive impairment and its mechanism of occurrence.

## 5 Conclusion

The results of the meta-analysis in this study showed a significant positive association between constipation and cognitive impairment, which was more significant in patients with Parkinson's disease. Based on the pathological mechanisms of PD-specific cholinergic phenotypes (characterized by hyposmia, weight loss, and rapid cognitive decline) and noradrenergic phenotypes (manifested as RBD, anxiety, etc.), constipation may accelerate cognitive decline through pathways such as α-synuclein propagation along the gut-brain axis and neuroinflammation. Therefore, in clinical practice, constipation should be considered a key indicator in the staged management of Parkinson's disease (42). Early identification and intervention for patients with constipation, especially those in the PD group, should be implemented, including comprehensive measures such as regular assessment of intestinal function, cholinergic modulation, and gut-targeted therapies, with the aim of delaying the progression of cognitive decline and optimizing overall clinical management. It is recommended to incorporate systematic constipation screening into the standard diagnostic and treatment protocols for PD, as this may represent an intervenable target for improving the progression of both motor and non-motor symptoms in patients.

## Data Availability

The original contributions presented in the study are included in the article/Supplementary material, further inquiries can be directed to the corresponding author.

## Supplementary material

Supplementary Table S1 Search strategy.

Supplementary Figure S1 OR and 95% CI for CI in patients with constipation in different study types.

Supplementary Figure S2 OR and 95% CI of constipation patients with different gender ratios.

Supplementary Figure S3 OR and 95% CI of constipation patients in different age groups.

Supplementary Figure S4 OR and 95% CI for CI in patients with constipation from different regions.

Supplementary Figure S5 Sensitivity analysis of the included studies.

Supplementary Figure S6 PB in the included studies.
