# Supplementary material for: Constipation and risk of cognitive impairment and dementia in adults: a systematic review and meta-analysis
Source: Front Neurol. 2025 Jun 4;16:1600952. doi: 10.3389/fneur.2025.1600952 (PMC12173855; doi:10.3389/fneur.2025.1600952)
Supplement: Supplementary Table S1 — Search strategy. [file Table_1.docx]

**Table S1** Search Strategy

**Cochrane**

| No. | Query | Results |
| --- | --- | --- |
| #11 | #10 AND #3 | 652 |
| #10 | #4 OR #5 OR #6 OR #7 OR #8 OR #9 | 87,090 |
| #9 | (alzheimer syndrome OR alzheimer disease* OR alzheimer dementia*):ti,ab,kw OR (senile dementia* OR alzheimer sclerosis* OR Alzheimer):ti,ab,kw OR (diffuse cortical sclerosis):ti,ab,kw | 14,973 |
| #8 | MeSH descriptor: [Alzheimer Disease] explode all trees | 5,452 |
| #7 | (dementia*):ti,ab,kw OR (amentia*):ti,ab,kw OR (demention):ti,ab,kw | 18,414 |
| #6 | MeSH descriptor: [Dementia] explode all trees | 9,658 |
| #5 | (cognitive dysfunction* OR cognitive disorder* OR cognitive impairment* OR cognitive decline*):ti,ab,kw OR (cognitive complaints OR cognitive defect* OR cognitive defici* OR cognitive difficult*):ti,ab,kw OR (cognitive disability OR cognitive disturbance OR cognitive problem* OR overinclusion OR response interference):ti,ab,kw | 70,615 |
| #4 | MeSH descriptor: [Cognitive Dysfunction] explode all trees | 4,205 |
| #3 | #1 OR #2 | 18,264 |
| #2 | (Colonic Inertia OR Dyschezia OR Constipated OR defecation disorder OR Constipation):ti,ab,kw OR (evacuation disorder* OR gastrointestinal transit OR gut transit OR slow transit):ti,ab,kw OR (hard stool* OR lumpy stool OR obstipation):ti,ab,kw | 18,264 |
| #1 | MeSH descriptor: [Constipation] explode all trees | 2,454 |

**Embase**

| No. | Query | Results |
| --- | --- | --- |
| #6 | #5 AND #1 | 6,088 |
| #5 | #2 OR #3 OR #4 | 767,086 |
| #4 | 'alzheimer disease'/exp OR 'alzheimer syndrome':ab,ti OR 'alzheimer disease*':ab,ti OR 'alzheimer dementia*':ab,ti OR 'senile dementia*':ab,ti OR 'alzheimer sclerosis*':ab,ti OR alzheimer:ab,ti OR 'diffuse cortical sclerosis':ab,ti | 319,920 |
| #3 | 'dementia'/exp OR dementia*:ab,ti OR amentia*:ab,ti OR demention:ab,ti | 512,157 |
| #2 | 'cognitive defect'/exp OR 'cognitive dysfunction*':ab,ti OR 'cognitive disorder*':ab,ti OR 'cognitive impairment*':ab,ti OR 'cognitive decline*':ab,ti OR 'cognitive complaints':ab,ti OR 'cognitive defect*':ab,ti OR 'cognitive defici*':ab,ti OR 'cognitive difficult*':ab,ti OR 'cognitive disability':ab,ti OR 'cognitive disturbance':ab,ti OR 'cognitive problem*':ab,ti OR overinclusion:ab,ti OR 'response interference':ab,ti | 715,656 |
| #1 | 'constipation'/exp OR 'colonic inertia':ab,ti OR dyschezia:ab,ti OR constipation:ab,ti OR constipated:ab,ti OR 'defecation disorder':ab,ti OR 'evacuation disorder*':ab,ti | 131,720 |

**Pubmed**

| No. | Query | Results |
| --- | --- | --- |
| #6 | (((((((((((((((((cognitive dysfunction[MeSH Terms])) OR (cognitive dysfunction*[Title/Abstract])) OR (cognitive disorder*[Title/Abstract])) OR (cognitive impairment*[Title/Abstract])) OR (cognitive decline*[Title/Abstract])) OR (cognitive complaints[Title/Abstract])) OR (cognitive defect*[Title/Abstract])) OR (cognitive defici*[Title/Abstract])) OR (cognitive difficult*[Title/Abstract])) OR (cognitive disability[Title/Abstract])) OR (cognitive disturbance[Title/Abstract])) OR (cognitive problem*[Title/Abstract])) OR (overinclusion[Title/Abstract])) OR (response interference[Title/Abstract])) OR ((((dementia[MeSH Terms]) OR (dementia*[Title/Abstract])) OR (amentia*[Title/Abstract])) OR (demention[Title/Abstract]))) OR ((((((((alzheimer disease[MeSH Terms]) OR (alzheimer syndrome[Title/Abstract])) OR (alzheimer disease*[Title/Abstract])) OR (alzheimer dementia*[Title/Abstract])) OR (senile dementia*[Title/Abstract])) OR (alzheimer sclerosis*[Title/Abstract])) OR (Alzheimer[Title/Abstract])) OR (diffuse cortical sclerosis[Title/Abstract]))) AND (((((((((((((constipation[MeSH Terms]) OR (Colonic Inertia[Title/Abstract])) OR (Dyschezia[Title/Abstract])) OR (Constipation[Title/Abstract])) OR (constipated[Title/Abstract])) OR (defecation disorder[Title/Abstract])) OR (evacuation disorder*[Title/Abstract])) OR (gastrointestinal transit[Title/Abstract])) OR (gut transit[Title/Abstract])) OR (slow transit[Title/Abstract])) OR (hard stool*[Title/Abstract])) OR (lumpy stool[Title/Abstract])) OR (obstipation[Title/Abstract])) | 671 |
| #5 | ((((((((((((((((cognitive dysfunction[MeSH Terms])) OR (cognitive dysfunction*[Title/Abstract])) OR (cognitive disorder*[Title/Abstract])) OR (cognitive impairment*[Title/Abstract])) OR (cognitive decline*[Title/Abstract])) OR (cognitive complaints[Title/Abstract])) OR (cognitive defect*[Title/Abstract])) OR (cognitive defici*[Title/Abstract])) OR (cognitive difficult*[Title/Abstract])) OR (cognitive disability[Title/Abstract])) OR (cognitive disturbance[Title/Abstract])) OR (cognitive problem*[Title/Abstract])) OR (overinclusion[Title/Abstract])) OR (response interference[Title/Abstract])) OR ((((dementia[MeSH Terms]) OR (dementia*[Title/Abstract])) OR (amentia*[Title/Abstract])) OR (demention[Title/Abstract]))) OR ((((((((alzheimer disease[MeSH Terms]) OR (alzheimer syndrome[Title/Abstract])) OR (alzheimer disease*[Title/Abstract])) OR (alzheimer dementia*[Title/Abstract])) OR (senile dementia*[Title/Abstract])) OR (alzheimer sclerosis*[Title/Abstract])) OR (Alzheimer[Title/Abstract])) OR (diffuse cortical sclerosis[Title/Abstract])) | 452,654 |
| #4 | (((((((alzheimer disease[MeSH Terms]) OR (alzheimer syndrome[Title/Abstract])) OR (alzheimer disease*[Title/Abstract])) OR (alzheimer dementia*[Title/Abstract])) OR (senile dementia*[Title/Abstract])) OR (alzheimer sclerosis*[Title/Abstract])) OR (Alzheimer[Title/Abstract])) OR (diffuse cortical sclerosis[Title/Abstract]) | 220,545 |
| #3 | (((dementia[MeSH Terms]) OR (dementia*[Title/Abstract])) OR (amentia*[Title/Abstract])) OR (demention[Title/Abstract]) | 279,916 |
| #2 | ((((((((((((((cognitive dysfunction[MeSH Terms])) OR (cognitive dysfunction*[Title/Abstract])) OR (cognitive disorder*[Title/Abstract])) OR (cognitive impairment*[Title/Abstract])) OR (cognitive decline*[Title/Abstract])) OR (cognitive complaints[Title/Abstract])) OR (cognitive defect*[Title/Abstract])) OR (cognitive defici*[Title/Abstract])) OR (cognitive difficult*[Title/Abstract])) OR (cognitive disability[Title/Abstract])) OR (cognitive disturbance[Title/Abstract])) OR (cognitive problem*[Title/Abstract])) OR (overinclusion[Title/Abstract])) OR (response interference[Title/Abstract]) | 179,081 |
| #1 | ((((((((((((constipation[MeSH Terms]) OR (Colonic Inertia[Title/Abstract])) OR (Dyschezia[Title/Abstract])) OR (Constipation[Title/Abstract])) OR (constipated[Title/Abstract])) OR (defecation disorder[Title/Abstract])) OR (evacuation disorder*[Title/Abstract])) OR (gastrointestinal transit[Title/Abstract])) OR (gut transit[Title/Abstract])) OR (slow transit[Title/Abstract])) OR (hard stool*[Title/Abstract])) OR (lumpy stool[Title/Abstract])) OR (obstipation[Title/Abstract]) | 40,008 |

**Web Of Science**

| No. | Query | Results |
| --- | --- | --- |
| #6 | #5 AND #1 | 1,072 |
| #5 | #4 OR #3 OR #2 | 817,711 |
| #4 | alzheimer disease (主题) OR Alzheimer disease OR alzheimer syndrome OR alzheimer disease* (主题) OR alzheimer dementia* OR senile dementia* OR alzheimer sclerosis* (主题) OR Alzheimer OR diffuse cortical sclerosis (主题) | 239,073 |
| #3 | dementia (主题) OR dementia* (主题) OR amentia* (主题) OR demention (主题) | 230,026 |
| #2 | cognitive dysfunction* OR cognitive disorder* OR cognitive impairment* (主题) OR cognitive decline* OR cognitive complaints OR cognitive defect* (主题) OR cognitive defici* OR cognitive difficult* OR cognitive disability OR cognitive disturbance (主题) OR cognitive problem* OR overinclusion OR response interference (主题) | 558,239 |
| #1 | Colonic Inertia OR Dyschezia OR Constipation (主题) OR dyschezia OR constipated OR defecation disorder OR evacuation disorder* (主题) OR gastrointestinal transit OR gut transit OR slow transit OR hard stool* (主题) OR lumpy stool OR obstipation (主题) | 48,320 |
