# Supplementary figures and images for: Constipation and risk of cognitive impairment and dementia in adults: a systematic review and meta-analysis
Source: Front Neurol. 2025 Jun 4;16:1600952. doi: 10.3389/fneur.2025.1600952 (PMC12173855; doi:10.3389/fneur.2025.1600952)

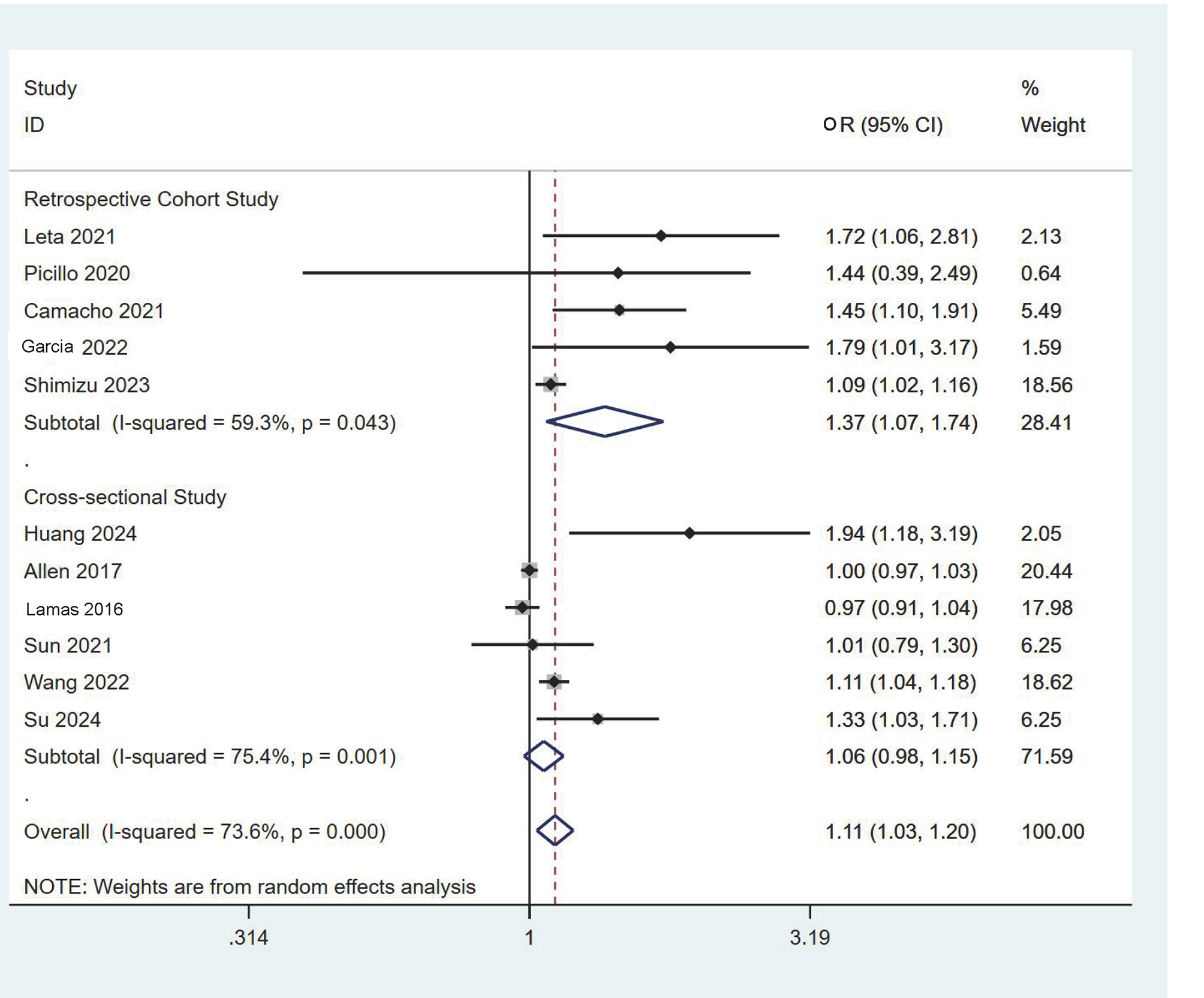

Supplement: Supplementary Figure S1 — OR and 95% CI for CI in patients with constipation in different study types. [file Image_1.tif]

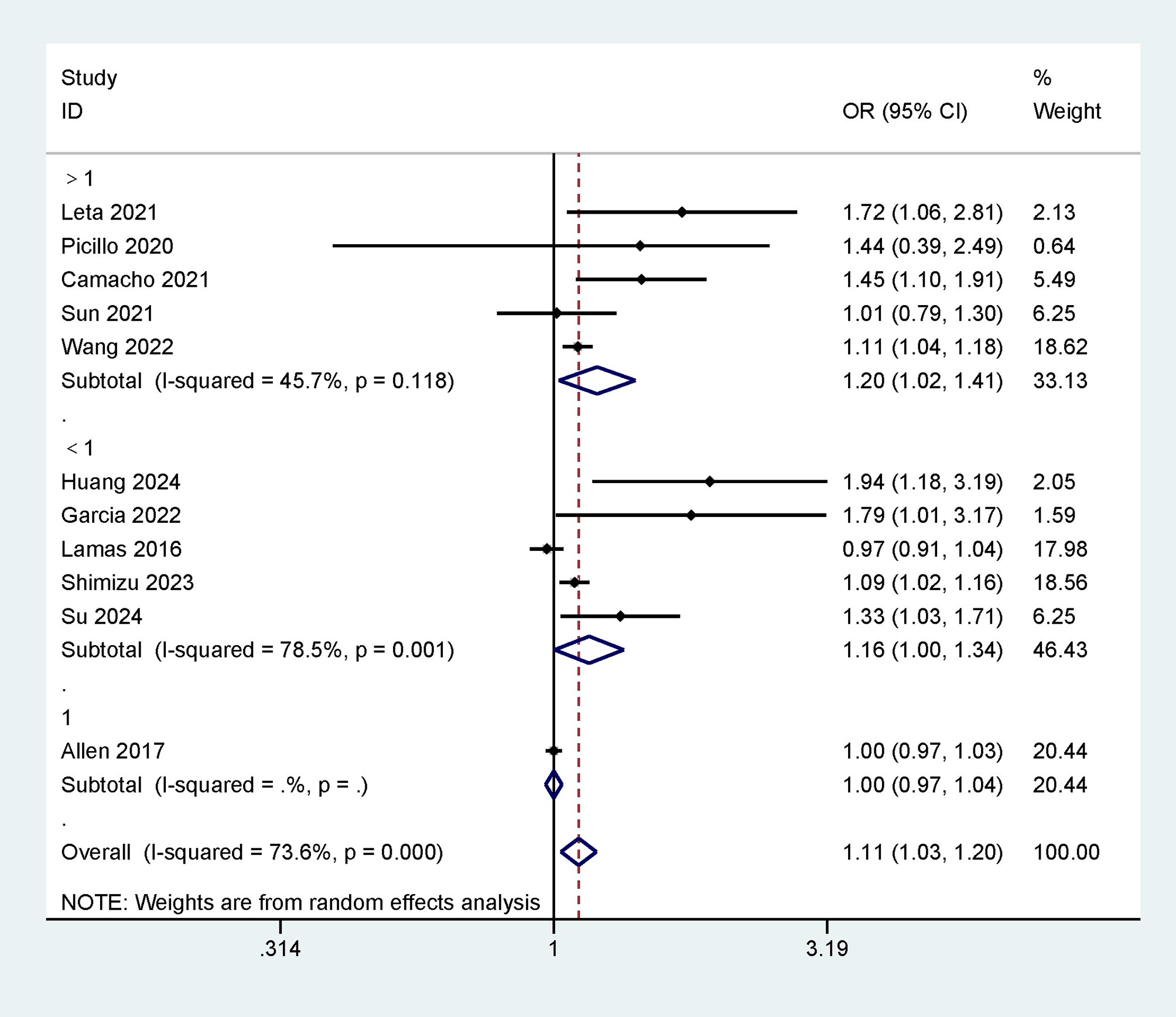

Supplement: Supplementary Figure S2 — OR and 95% CI of constipation patients with different gender ratios. [file Image_2.tif]

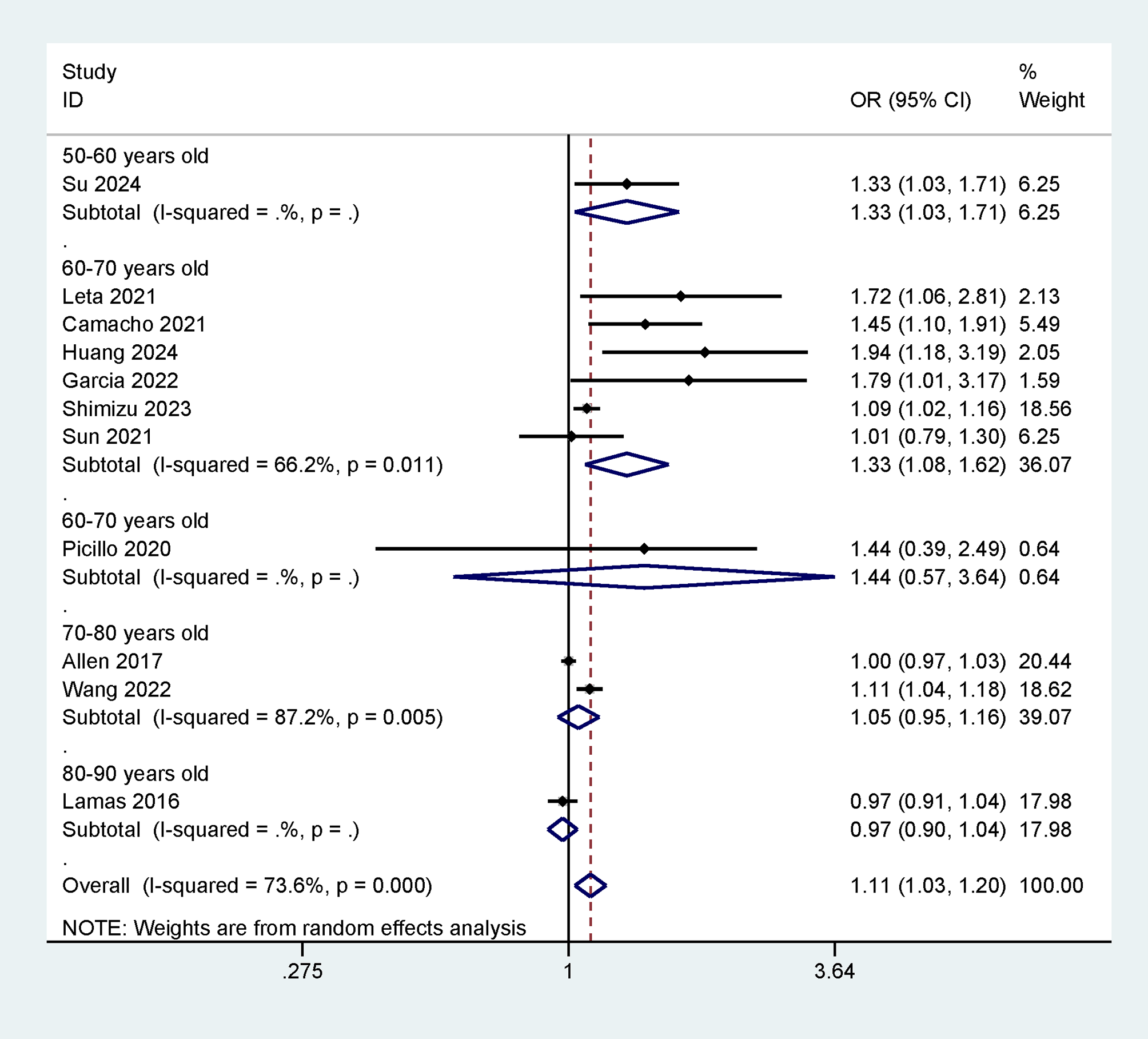

Supplement: Supplementary Figure S3 — OR and 95% CI of constipation patients in different age groups. [file Image_3.tif]

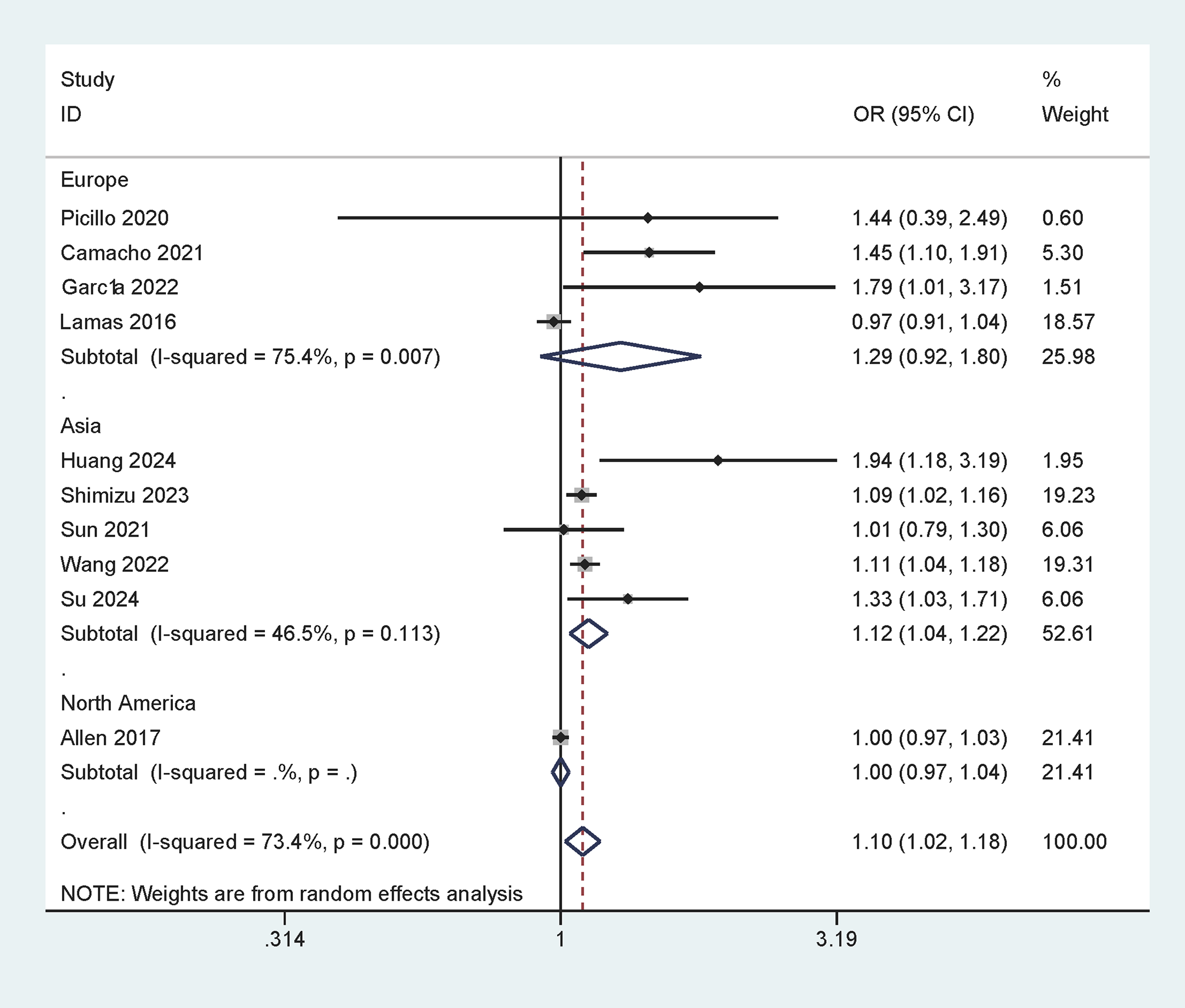

Supplement: Supplementary Figure S4 — OR and 95% CI for CI in patients with constipation from different regions. [file Image_4.tif]

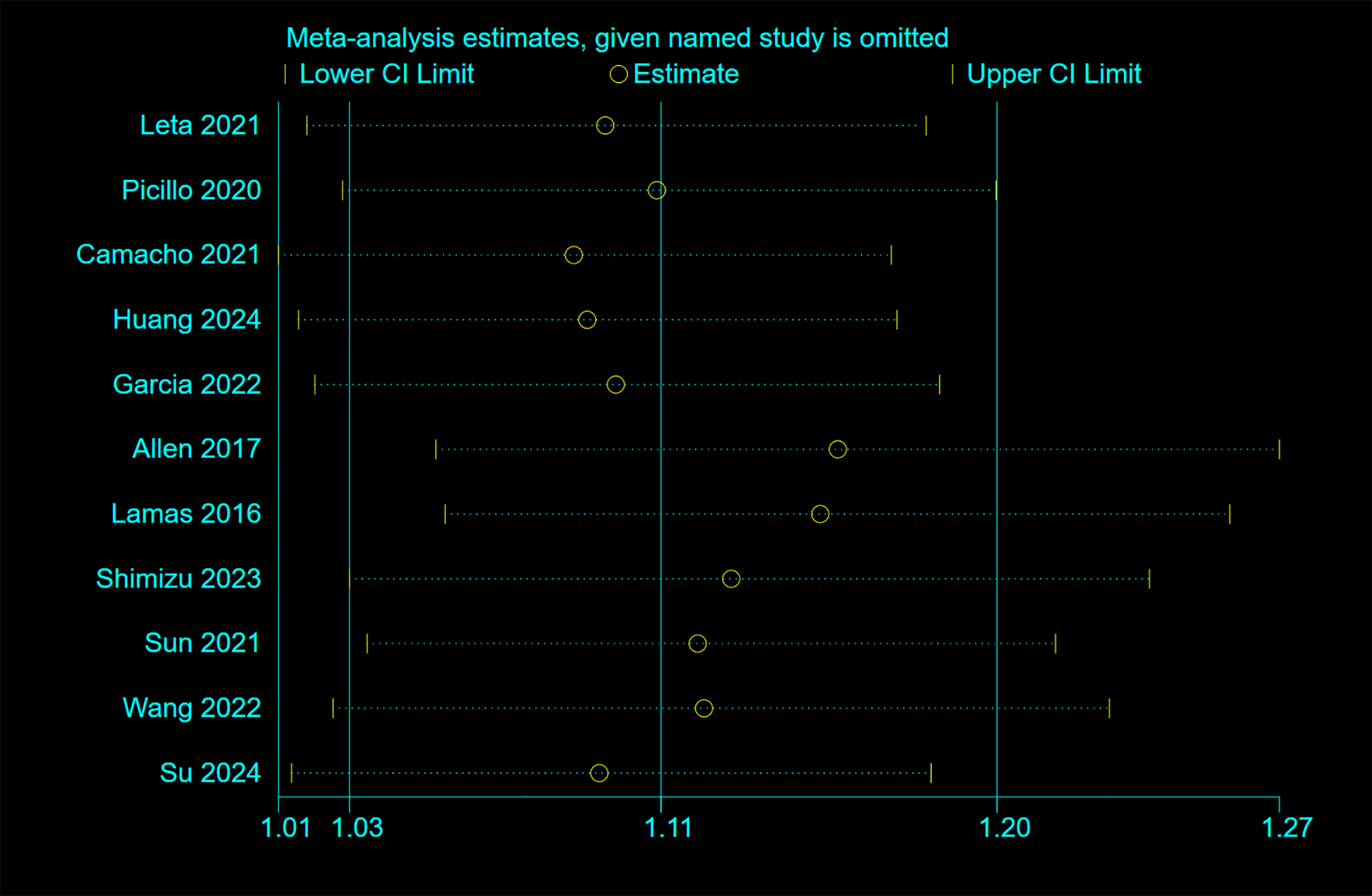

Supplement: Supplementary Figure S5 — Sensitivity analysis of the included studies. [file Image_5.tif]

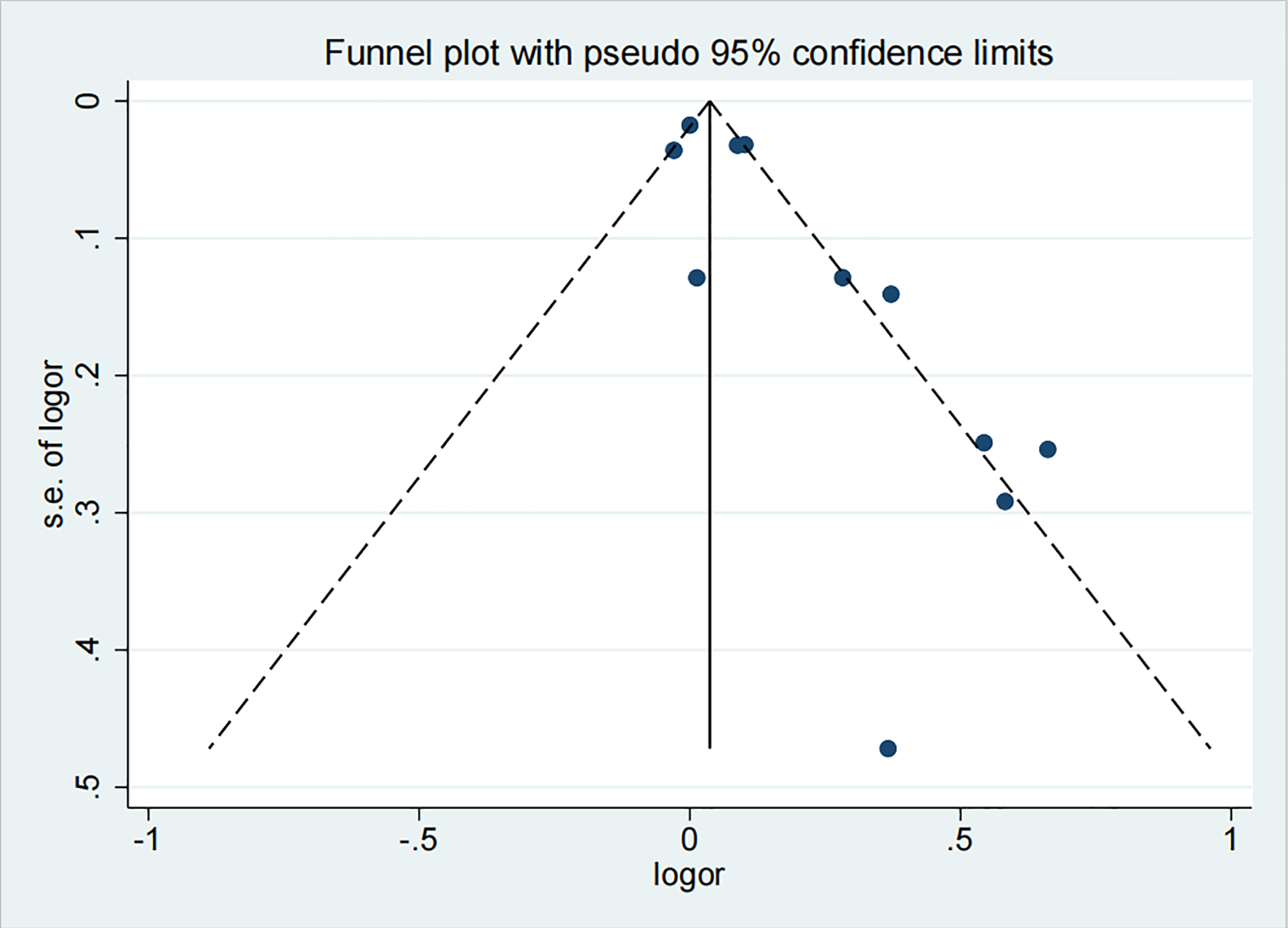

Supplement: Supplementary Figure S6 — PB in the included studies. [file Image_6.tif]
